# Pretreatment with *Bifidobacterium longum* BAA2573 ameliorates dextran sulfate sodium (DSS)-induced colitis by modulating gut microbiota

**DOI:** 10.3389/fmicb.2023.1211259

**Published:** 2023-06-06

**Authors:** Qiong Lin, Wu-Juan Hao, Ren-Min Zhou, Cui-Lan Huang, Xu-Yang Wang, Yan-Shan Liu, Xiao-Zhong Li

**Affiliations:** ^1^Nephrology and Immunology Department, Children's Hospital of Soochow University, Suzhou, Jiangsu, China; ^2^Department of Digestive, Affiliated Children's Hospital of Jiangnan University, Wuxi, Jiangsu, China; ^3^Nanjing Medical University, Nanjing, Jiangsu, China; ^4^Department of Pediatric Laboratory, Affiliated Children's Hospital of Jiangnan University, Wuxi, Jiangsu, China

**Keywords:** bifidobacterium longum, DSS-induced colitis, gut microbiota, metabolites, inflammatory bowel disease, probiotics

## Abstract

**Objectives:**

Inflammatory bowel disease (IBD) is a chronic lifelong inflammatory disease. Probiotics such as *Bifidobacterium longum* are considered to be beneficial to the recovery of intestinal inflammation by interaction with gut microbiota. Our goals were to define the effect of the exclusive use of BAA2573 on dextran sulfate sodium (DSS)-induced colitis, including improvement of symptoms, alleviation of histopathological damage, and modulation of gut microbiota.

**Methods:**

In the present study, we pretreated C57BL/6J mice with *Bifidobacterium longum* BAA2573, one of the main components in an over-the-counter (OTC) probiotic mixture BIFOTO capsule, before modeling with DSS. 16S rDNA sequencing and liquid chromatography–tandem mass spectrometry (LC-MS/MS)-based non-targeted metabolomic profiling were performed with the collected feces.

**Results:**

We found that pretreatment of *Bifidobacterium longum* BAA2573 given by gavage significantly improved symptoms and histopathological damage in DSS-induced colitis mice. After the BAA2573 intervention, 57 genera and 39 metabolites were significantly altered. Pathway enrichment analysis demonstrated that starch and sucrose metabolism, vitamin B6 metabolism, and sphingolipid metabolism may contribute to ameliorating colitis. Moreover, we revealed that the gut microbiome and metabolites were interrelated in the BAA2573 intervention group, while Alistipes was the core genus.

**Conclusion:**

Our study demonstrates the impact of BAA2573 on the gut microbiota and reveals a possible novel adjuvant therapy for IBD patients.

## 1. Introduction

Inflammatory bowel disease (IBD) is a chronic inflammatory disease of gastrointestinal tissues that is prone to recurrent attacks. IBD includes two main types, namely ulcerative colitis (UC) and Crohn's disease (CD) (Flynn and Eisenstein, 2019). Severe cases suffer from intestinal perforation, intestinal fistula, perianal abscess, arthritis, or other extraintestinal manifestations (Rogler et al., 2021). Moreover, most IBD patients also face the risk of a weakened immune system and even bowel cancer (Keller et al., 2019). The incidence and prevalence of IBD, especially pediatric inflammatory bowel disease (PIBD) in newly industrialized countries, especially some of them in Asia, have increased sharply in recent years (Ng et al., 2017; Kuenzig et al., 2022). The pathogenesis of IBD is closely related to host genetic susceptibility, intestinal flora, environmental factors, and immune disorders (Li et al., 2015; Gao et al., 2021). Currently, there is no cure for IBD, but treatment can help manage symptoms and reduce inflammation. However, the long-term efficacy and side effects of current drugs are unpredictable since they could be affected by several factors such as disease severity and individual response to treatment, leading to serious economic burden and social pressure on patients, families, and society. New targets for the prevention and treatment of IBD are urgently needed (D'Haens et al., 2022).

Intestinal microbiota, the so-called the biological barrier to the intestine, which participates in the physiological activities of the host by maintaining immune balance and producing beneficial metabolites, is closely related to the onset of IBD (Li et al., 2015; Hu et al., 2022). Studies have found that the composition and metabolites of intestinal flora in IBD patients are disordered, resulting in further damage to the intestinal barrier (Lin et al., 2023), decreased expression of tight junction proteins (Shi et al., 2019) and antimicrobial peptides (Gubatan et al., 2021; Liu Z. et al., 2022), and dysfunction of the intestinal immune response (Dong F. et al., 2022). Probiotics are widely used in the adjuvant treatment of diseases because of their high safety and good intestinal tolerance. Among them, *Bifidobacterium longum* is one of the most abundant microorganisms in the gut of infants and adults, which even can be transformed from mom to offspring by breastfeeding (Qi et al., 2022a). Therefore, it is critical for the development of the immune system and is the preferred choice of probiotics (Qi et al., 2022b). Studies have shown that *Bifidobacterium longum* can reduce the expression of inflammatory cytokine (Chen et al., 2016; Singh et al., 2020), balance intestinal immunity (Roselli et al., 2009; Yao et al., 2011), bring down reactive oxygen species (ROS) level (Wang et al., 2021), repair and strengthen the intestinal mucosal barrier, and regulate gut microbiota (Ni et al., 2023), consequently alleviating the symptoms of acute colitis and improving IBD clinically (Miele et al., 2009). Even in infants, *Bifidobacterium longum* supplementation causes few gastrointestinal side effects or dysfunctions of the liver and kidneys (Manzano et al., 2017). Different types of probiotics have different effects (Rodríguez-Nogales et al., 2018; Zhao et al., 2022). The mechanism of certain *Bifidobacterium longum* in the treatment of IBD needs to be further explored.

BIFOTO capsule is an over-the-counter (OTC) probiotic mixture and is mainly used to treat gastrointestinal dysfunction caused by intestinal flora imbalance. *Bifidobacterium longum* BAA2573 (BAA2573), *Lactobacillus acidophilus*, and *Enterococcus faecalis* are the main components (Editorial Board of Chinese Journal of Digestion, 2022). Previous studies have demonstrated that the probiotic cocktail BIFICO could inhibit the inflammatory response in *H. pylori*-induced gastritis (Yu et al., 2015), ameliorate colitis-associated cancer in mice (Song et al., 2018), enhance the curative effect and reduce adverse reactions of mesalazine for UC patients (Jiang et al., 2020), and reduce the recurrence rate of UC (Chen M. Y. et al., 2019), but the properties of the single strain remain unknown.

Studies have shown that DSS-induced colitis was a rapid and practical model for the study of IBD (Eichele and Kharbanda, 2017). DSS in drinking water would directly act on the colon and rectum, damage intestinal epithelial cells, destroy the intestinal barrier, and induce acute colitis (Wirtz et al., 2017; Katsandegwaza et al., 2022). In the present study, we selected BAA2573 for further investigation due to its wide application and few side effects. We investigated symptoms, histopathological damage, compositional changes and interplay in the commensal microbiota, and metabolites of the mouse colon to better understand the effectiveness and the underlying mechanism in the treatment of DSS-induced colitis. This study demonstrates the potential impact of BAA2573 on colitis and provides theoretical support for the application of this probiotic strain in IBD.

## 2. Materials and methods

### 2.1. Animal experiments

**Figure 1:**
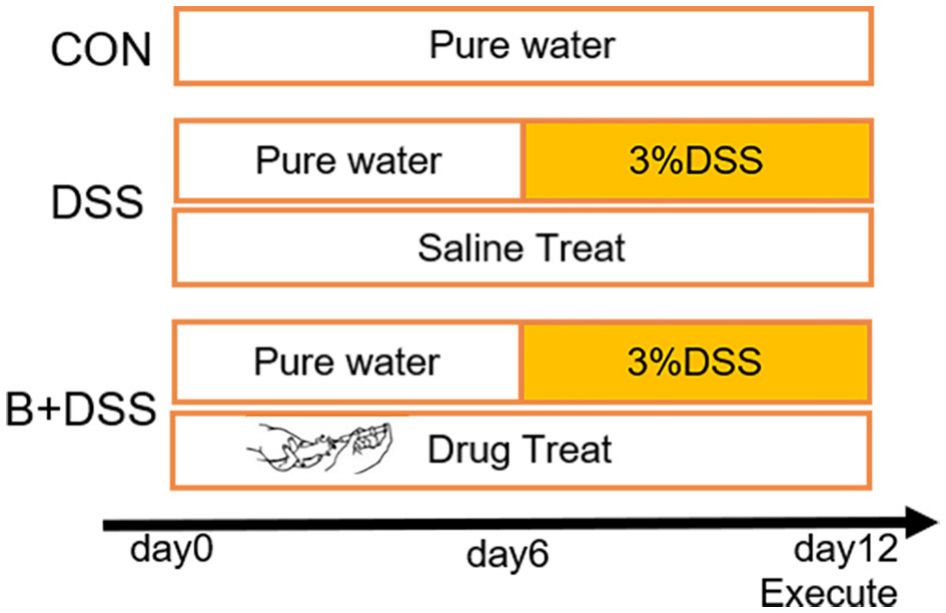
Experimental design and procedure. CON group: normal diet and water; DSS group: colitis model (3% DSS in drink water from day 6) + normal saline (0.2 ml/day); B+DSS group: colitis model (3% DSS in drink water from day 6) + *Bifidobacterium longum* BAA2573 (~1 × 10^10^ CFU/kg/day).

The experimental procedure was approved by the Ethics Committee of Wuxi Children's Hospital (WXCH2022-10-073), and all operations met the National Institutes of Health guidelines. We purchased 6-week-old wild-type C57BL/6J mice from Changzhou Cavens Laboratory Animal Co. Ltd. through BioMart (Changzhou, China) and kept them in the animal room of Wuxi People's Hospital (room temperature: 20 ± 2°C, 12-h−12-h day and night cycle). After 7 days of adaptive feeding with normal diet and water, mice were randomly divided into three groups (*n* = 8 per group): control group (CON), 3% DSS group (DSS, molecular weight: 35,000–50,000, purchased from MP Biomedicals), and *Bifidobacterium longum* intervention group (B+DSS, BAA2573 was purchased from Shanghai Sine Company). The modeling process is shown in Figure 1. Referring to previous animal experiments (Dong J. et al., 2022), the B+DSS group was given 0.2 ml of bacterial solution at 10 a.m. daily by oral gavage (about 1 × 10^10^ CFU/kg), while the DSS group was given 0.2 ml of normal saline. The weight and stool characteristics were recorded daily to evaluate whether the model was established. On day 12, all mice were sacrificed by cervical dislocation after anesthesia. The whole colon tissue was photographed under sterile conditions to record the length. A total volume of 1 ml of intestinal contents were retained by lavaging with normal saline and then stored at −80°C for examination. The colonic tissue was fixed in 4% paraformaldehyde or stored at −80°C for examination. The disease activity index (DAI) score was calculated daily by weight loss, stool consistency, and stool bleeding to assess the severity of colitis in each group.

### 2.2. Histopathological analysis

We embedded 4% paraformaldehyde-fixed colonic tissue in paraffin, prepared it in a 5-μm section, and then stained it with hematoxylin and eosin (H&E) according to the product manual (purchased from Biyuntian, catalog number C0105S). Two pathologists finished double-blind scoring to assess tissue damage. The scoring criteria were referred to three parts: (a) epithelial impairment, (b) ulcer, and (c) inflammatory cell infiltration. For score (a): normal: 0; goblet cell reduction in less than one-third area: 1; goblet cell reduction in more than one-third area: 2; loss of crypt: 3; polypoid regeneration: 4. For score (b):0: 0; 1: 1; 2: 2; 3: 3; and over 3: 4. For score (c): normal: 0; around the crypts: 1; infiltration to the muscularis mucosa: 2; widespread infiltration of muscularis mucosa or with mucosal thickening: 3; infiltration to the submucosa: 4.

### 2.3. 16S rDNA sequencing

To determine pretreatment with *Bifidobacterium longum* BAA2573-induced gut microbiota alterations, DNA from fecal samples was extracted by hexadecyltrimethylammonium bromide (CTAB). In polymerase chain reaction (PCR) amplification, universal primers 515F (5′- GTGYCAGCMGCCGCGGTAA-3′) and 806R (5′- GGACTACHVGGGTWTCTAAT-3′) were used to target 16S rRNA gene V4 hypervariable regions. The purification of PCR products was performed by AMPure XP beads (Beckman Coulter Genomics, Danvers, MA, USA) and quantification by Qubit (Invitrogen, USA). The size and quantity of the amplicon library were assessed on Agilent 2100 Bioanalyzer (Agilent, USA) and with the Library Quantification Kit for Illumina (Kapa Biosciences, Woburn, MA, USA), respectively. The libraries were sequenced on the NovaSeq PE250 platform. After being primer-truncated, the raw paired-end reads were assigned to samples and merged using FLASH. To obtain the high-quality clean tags, the filtration was performed using fqtrim (v0.94) and Vsearch software (v2.3.4) successively. Next, clean data were dereplicated and denoised into amplicon sequence variation (ASVs) using Divisive Amplicon Denoising Algorithm (DADA2) before alpha and beta diversity analyses (Callahan et al., 2016).

### 2.4. Liquid chromatography–tandem mass spectrometry (LC-MS/MS)-based non-targeted metabolomic profiling

Metabolic extracts were obtained from stool samples. Then, LC-MS/MS analyses were performed using the UHPLC system (Vanquish, Thermo Fisher Scientific) with an ultra-performance liquid chromatography ethylene bridged hybrid (UPLC BEH) Amide column (2.1 mm × 100 mm, 1.7 μm) coupled to Orbitrap Exploris 120 mass spectrometer (Orbitrap MS, Thermo Fisher Scientific). The mobile phase consisted of 25 mmol/L ammonium acetate and 25 ammonia hydroxide in water (pH = 9.75) (A) and acetonitrile (B). The Orbitrap Exploris 120 mass spectrometer was used for its ability to acquire MS/MS spectra on information-dependent acquisition (IDA) mode in the control of the acquisition software (Xcalibur, Thermo Fisher Scientific). In this mode, the acquisition software continuously evaluates the full scan MS spectrum. Next, raw data were converted to the mzXML format using ProteoWizard and processed by an in-house program, which was developed based on R package XCMS (V.3.2). Subsequently, processes (Alseekh et al., 2021), such as peak detection, extraction, alignment, and integration, were performed by this in-house program. Then, metabolite annotation was finished using an in-house MS2 database. The cutoff for annotation was set at 0.3.

### 2.5. Statistical analysis

Data of weight, DAI score, and histopathological score were expressed as mean ± SD. Differences between the three groups were calculated using the one-way ANOVA (Tukey's tests). Alpha (α) diversity and beta (β) diversity were calculated with QIIME2. Feature abundance was normalized using the relative abundance of each sample. α diversity was applied to analyzing the complexity of species diversity for a sample through Chao 1 and Shannon indexes. Differences between groups were calculated using the Kruskal–Wallis test. β-diversity analysis is performed by principal coordinate analysis (PCoA). The relative abundances at the phylum or genus level between every two groups were analyzed *via* Student's *t*-test. LDA effect size (LefSe) analysis was used to compare the microbial compositions between the DSS and B+DSS groups (LefSe >3). To find significantly changed metabolites between groups, we used supervised orthogonal projections to latent structures discriminate analysis (OPLS-DA) in this study. Meanwhile, 200 times permutations and the permutation test were further conducted to check the robustness and predictive ability of the OPLS-DA model. Furthermore, the value of variable importance in the projection (VIP) of the first principal component in OPLS-DA analysis was achieved. Significantly altered metabolites referred to those with VIP>1, *P* < 0.05, fold change (FC)>2 or <0.2 (Student's *t*-test). Kyoto Encyclopedia of Genes and Genomes (KEGG) along with the MetaboAnalyst database was applied to pathway enrichment analysis. The Pearson correlation coefficient was used to evaluate the correlation between differentially abundant metabolites and microbiota (*r* > 0.4). A *P*-value of < 0.05 was considered to be statistically significant. The graphs were drawn by the R package (v3.5.2). The statistical analysis was generated with GraphPad Prism 9.3 software (GraphPad Inc., La Jolla, CA, USA).

## 3. Results

### 3.1. *Bifidobacterium longum* BAA2573 significantly improved symptoms and histopathological damage in DSS-induced colitis mice

**Figure 2:**
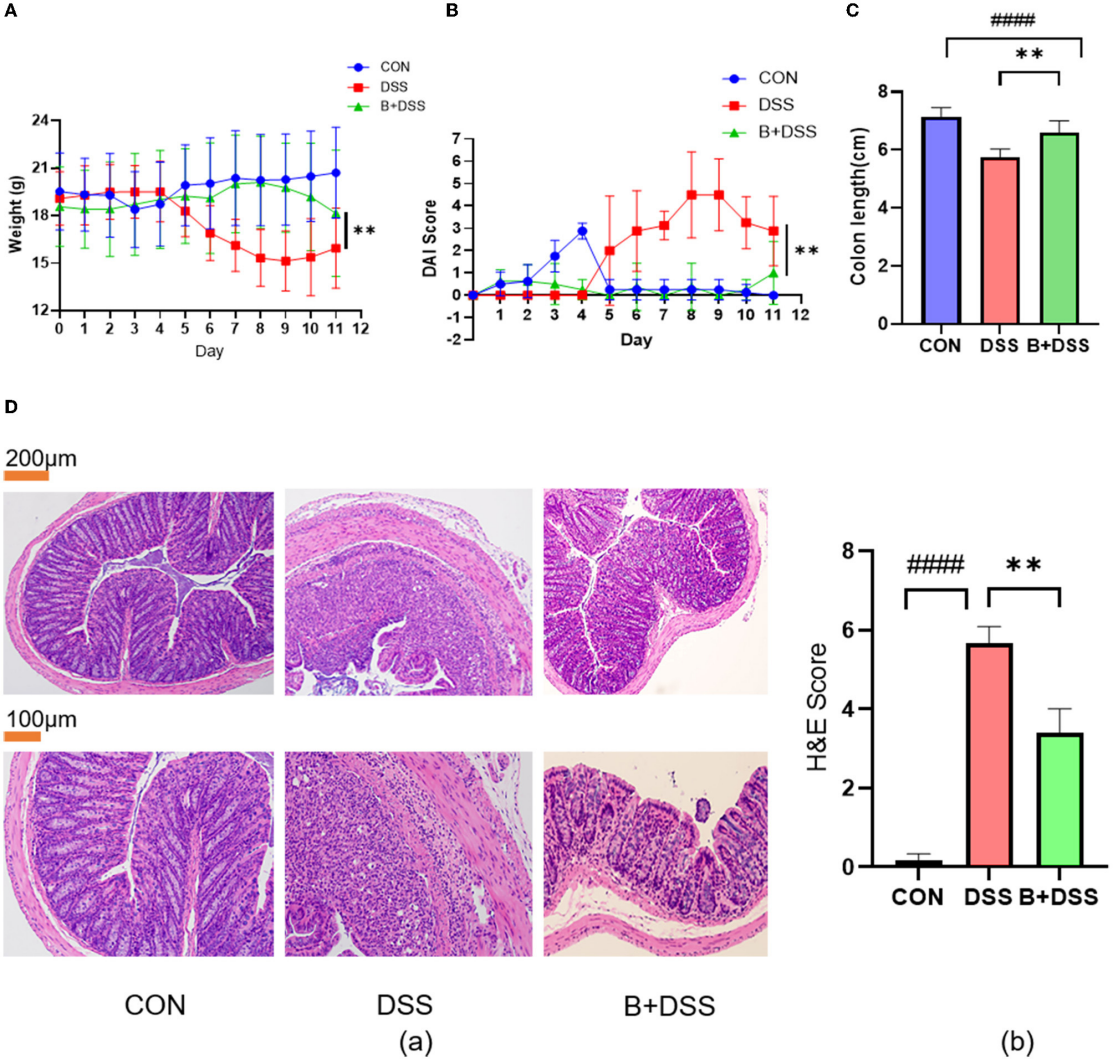
*Bifidobacterium longum* BAA2573 improved symptoms and histopathological damage in DSS-induced colitis mice. **(A, B)** The body weight and DAI score of the mice were assessed throughout the experiment. **(C)** The colon length of each group was measured. **(D)** (a) The distal colon was stained with H&E (neutrophilic infiltrates, absence of crypt structures, and even transmural inflammation in the DSS group; less neutrophil infiltrations and preserved crypt structures in the B+DSS group). (b) Histopathological score. The data are expressed as the means ± SDs (CON group n = 6, DSS group n = 6, B+DSS group n =6). The ^*^ symbol indicates any group compared with the DSS group; ^**^P < 0.01. The ^#^ symbol indicates any group compared with the CON group; ^####^*P* < 0.0001.

Weight loss, DAI scores, and colon length reflect the severity of damage in DSS-induced colitis. Compared to the initial weight at day 0, mice in the DSS group exhibited significant weight loss relative to another two groups (Figure 2A), while mice in the B+DSS group showed a mild decline after modeling, indicating that pretreatment with BAA2573 significantly alleviated weight loss caused by DSS (*p* < 0.05).

Similarly, DSS treatment remarkably increased the DAI scores, and pretreatment with BAA2573 reduced DAI scores after DSS modeling (Figure 2B). After 6 days of DSS exposure, the colons in the DSS group were significantly shortened. BAA2573 exhibited a distinct effect in increasing the length of the colon (*p* < 0.05) (Figure 2C). The photographs of the colon tissue are shown in Supplementary Figure 1. Taken together, these data indicated that pretreatment of *Bifidobacterium longum* BAA2573 significantly improved colitis-related parameters.

We further evaluated the effect of BAA2573 on alleviating DSS-induced colitis by the histological score of colon sections. As shown in Figure 2Da, the colon section of the DSS group exhibited extensive colonic damages, including neutrophilic infiltrates, absence of crypt structures, and even transmural inflammation, while none of these pathological features were found in the CON group. Compared with the DSS group, administration of BAA2573 resulted in fewer neutrophil infiltrations, preserved crypt structures in the colon sections, and decreased the histological scores with significant differences (*P* < 0.05) (Figure 2Db).

### 3.2. *Bifidobacterium longum* BAA2573 alleviated gut microbiota imbalance in DSS-induced colitis mice

**Figure 3:**
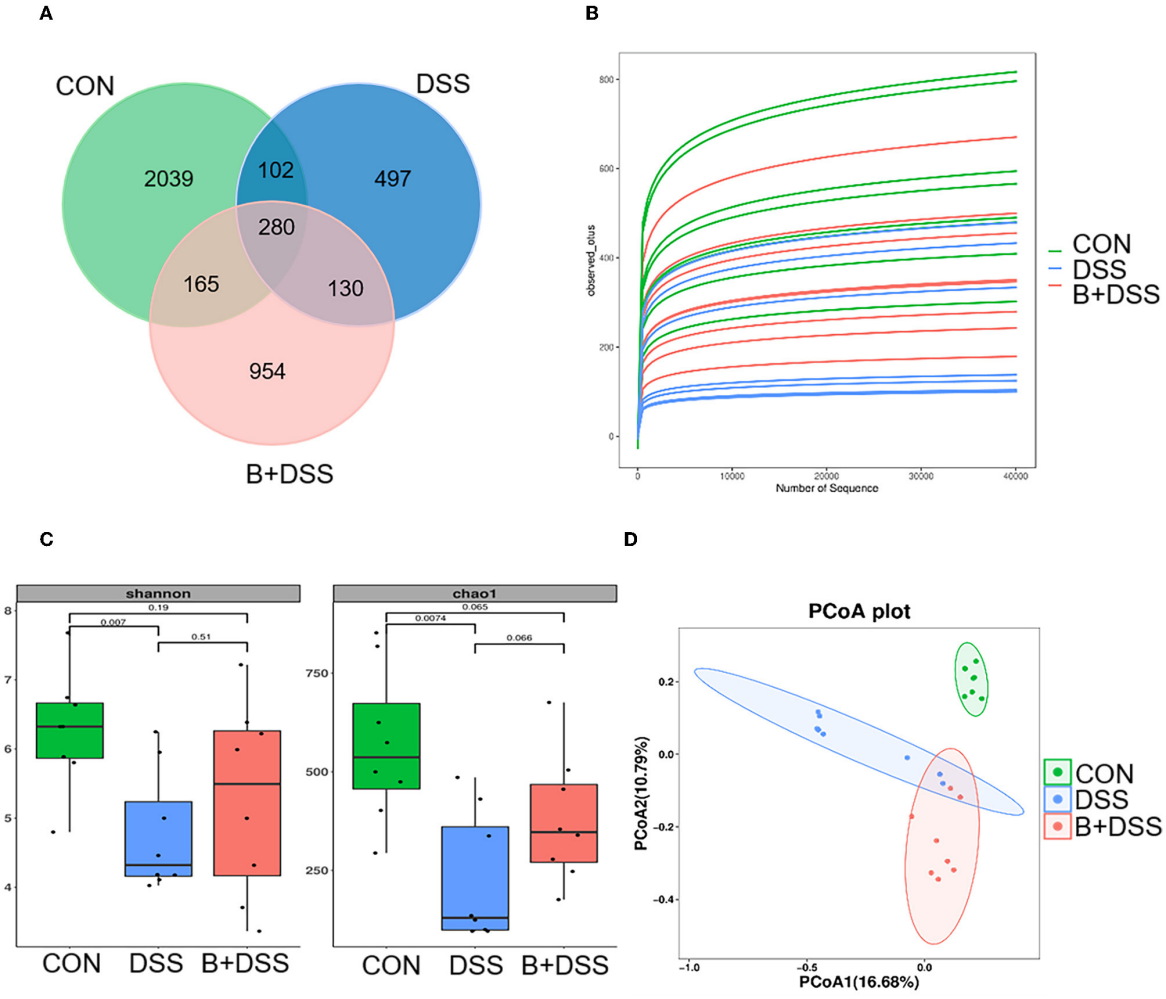
BAA2573 changes the structure of gut microbiota in DSS-induced colitis (n = 8). **(A)** A Venn diagram of ASVs in each group. **(B)** Rarefaction curve. **(C)** α-diversity analysis, including Shannon index and Chao 1 index. A *P*-value was marked directly in the figure. **(D)** β-diversity analysis was performed by principal coordinate analysis (PCoA).

Sequencing of 16S rDNA genes was performed to delineate changes in the gut microbiota composition. A total of 2039, 497, and 954 microbe ASVs were detected in the CON, DSS, and B+DSS groups, respectively (Figure 3A). A rarefaction curve based on the observed species specified that the sequencing data were sufficient to detect all species in the samples (Figure 3B). α-diversity analysis demonstrated that mice in the B+DSS group exhibited increased intestinal flora richness and diversity compared with the DSS group (Figure 3C). β-diversity represents the similarity of microbial composition among different groups, which showed that the similarity between the DSS group and the B+DSS group was observed only in a few samples (Figure 3D). A great difference in microbial composition between the CON group and the DSS group was observed with α- and β-diversity analyses, indicating that treatment with DSS disrupted homeostasis of the intestinal flora (Figures 3C, D).

**Figure 4:**
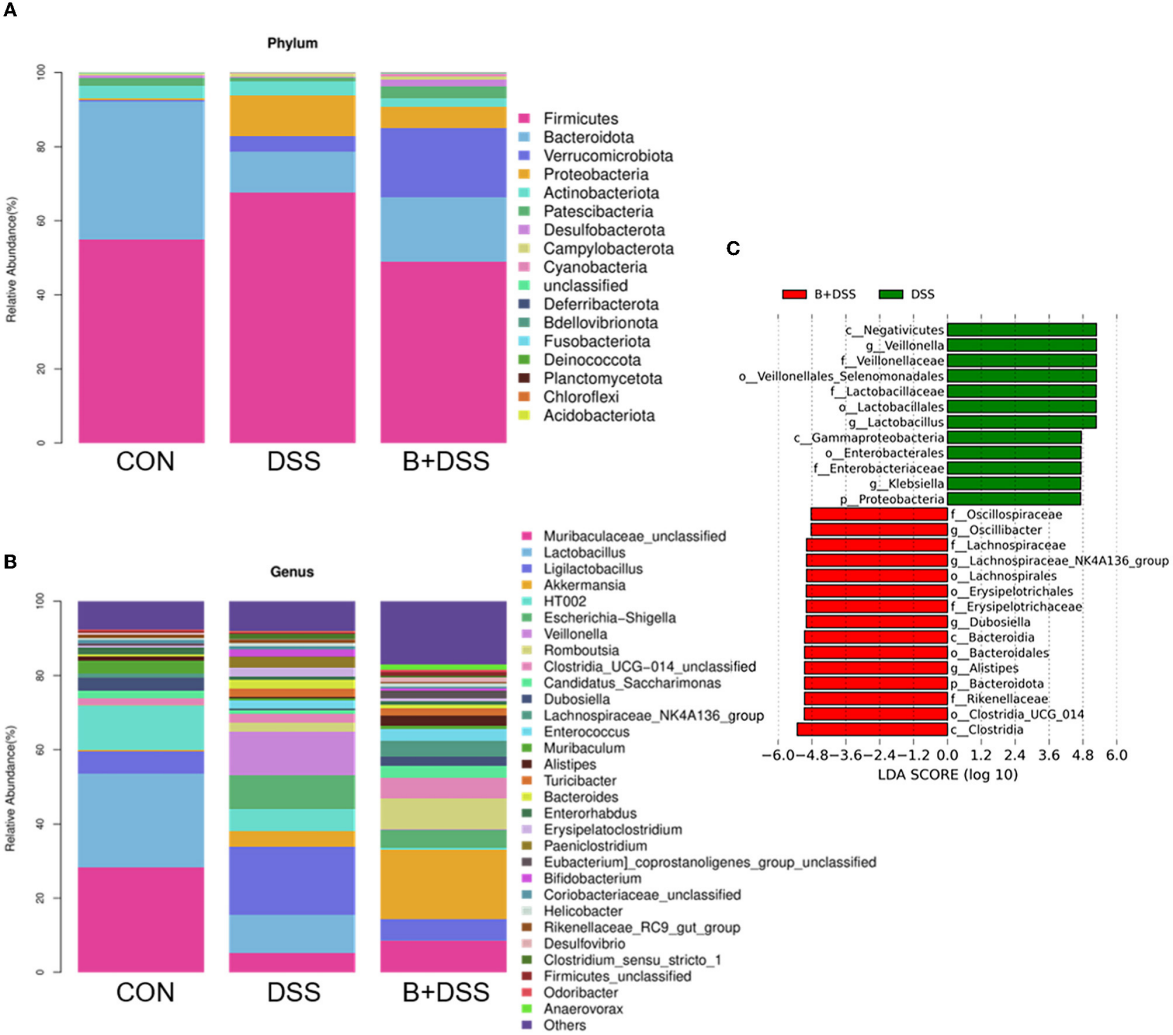
BAA2753 changes the composition of gut microbiota in DSS-induced colitis (*n* = 8). **(A)** Relative abundance of taxa at the phylum levels. **(B)** Relative abundance of taxa at the gene levels. **(C)** Linear discriminant analysis (LDA) score for predominant microbiota between the DSS and B+DSS groups.

The relative abundance of microbiota at the phylum levels was calculated in each group (Figure 4A). Compared with the DSS group, Bacteroidetes and Patescibacteria increased, and Firmicutes, Proteobacteria, and Actinobacteriota decreased after BAA2573 administration. A further subdivision at the genus level suggested a marked increase in *Klebsiella* (*P* = 0.006) and *Veillonella* (*P* = 0.016) and a decrease in *Candidatus_Saccharimonas* (*P* = 0.026), *Dubosiella* (*P* = 0.036), *Lachnospiraceae_NK4A136_group* (*P* = 0.006), *Lachnospiraceae_UCG-006* (*P* = 0.003), and *Alistipes* (*P* = 0.006) in the DSS group, and this dysbiosis was altered by BAA2573 (Figure 4B). A total of 57 genera were identified as significantly discriminative in the abundance between the B+DSS and DSS groups. To further determine the specific predominant bacteria associated with the pretreatment of BAA2573, LDA effect size (LefSe) analysis was used to compare the microbial compositions between the DSS and B+DSS groups. Among them, g_*Lactobacillus*, g_*Veillonella*, and g_*Klebsiella* were the main taxa enriched in the DSS group, while o_*Clostridia_UCG_014*, g_*Lachnospiraceae_NK4A136_group*, g_*Alistipes*, g_*Dubosiella*, and g_*Oscillibacter* were more abundant in the B+DSS group (Figure 4C).

### 3.3. *Bifidobacterium longum* BAA2573 altered abundant metabolites in DSS-induced colitis mice

**Figure 5:**
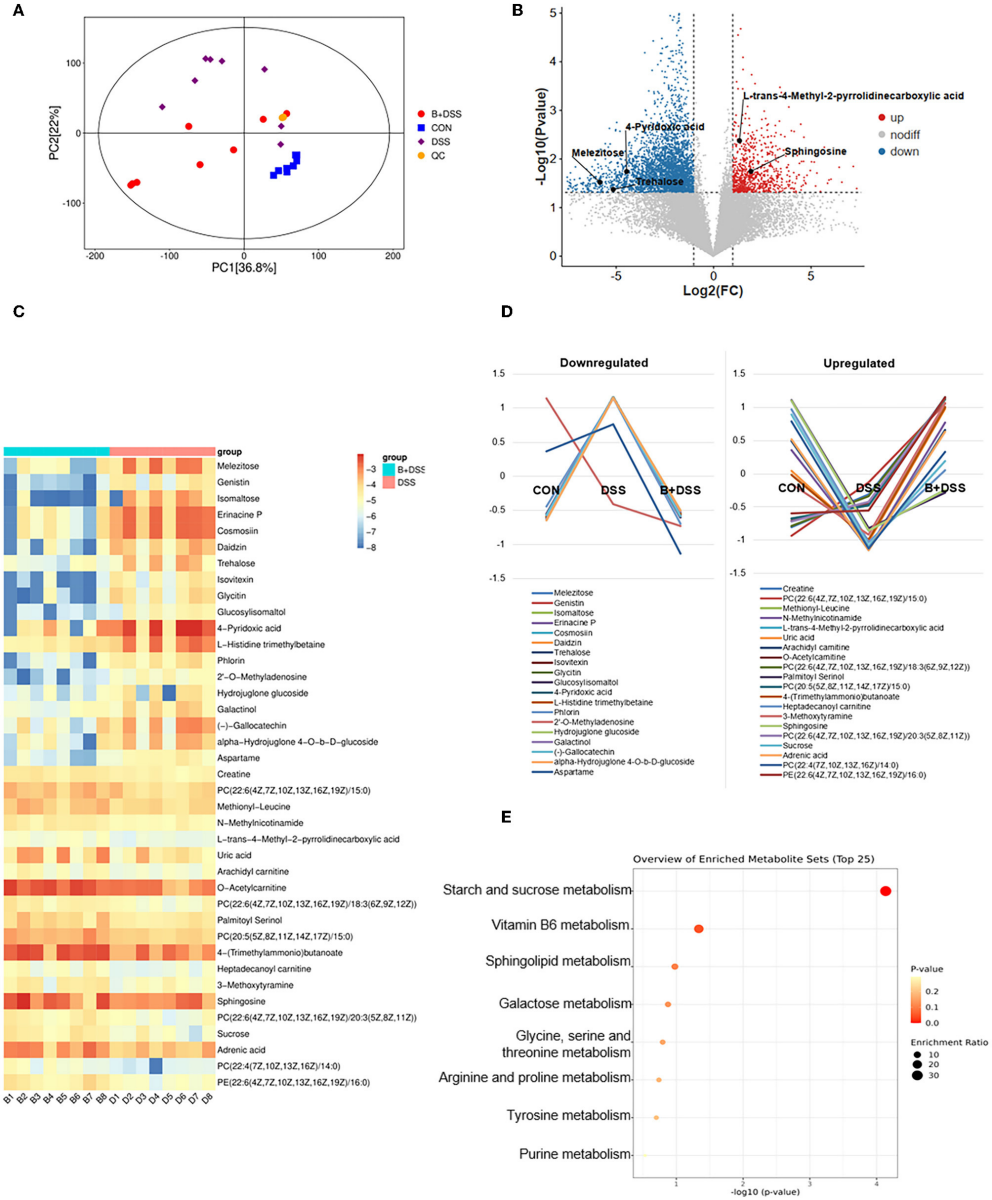
Pretreatment of BAA2753 altered metabolites of the colon in DSS-induced colitis. **(A)** Orthogonal partial least-squares discriminant analysis (OPLS-DA) in three groups and the quality control (QC) group. **(B)** The overall distribution of metabolites between the B+DSS and DSS groups was illustrated with a volcano plot. The vertical dashed lines indicate the threshold for the 2-fold abundance difference. The horizontal dashed line indicates the *p* = 0.05 threshold. Comparisons between the two groups were performed using Student's *t*-test. Metabolites with significant changes are presented in red (upregulated) or blue (downregulated). **(C)** Heatmap of fecal differential metabolites between the B+DSS and DSS groups after BAA2573 pretreatment. **(D)** K-means analysis was performed to delineate the trends in the relative concentrations of 39 metabolites in three groups. **(E)** KEGG pathway enrichment analysis.

LC-MS/MS-based non-targeted metabolomic profiling with collected stool samples from three groups was performed to investigate the differences in metabolic extracts. Univariate and multivariate analyses were used to screen out differential metabolites. The OPLS-DA model indicated the metabolic differences among the three groups (Figure 5A). The permutation test showed that the OPLS-DA model was not overfitting and had good validity (Supplementary Figure 2A). Volcano plots show the results of comparisons of metabolites between the CON and DSS groups (Supplementary Figure 2B) and the DSS and B+DSS groups (Figure 5B); meanwhile, altered expression levels were visualized with different colors.

With the inclusion criteria of VIP >1, FC ≥2 or <0.2, and P<0.05, 234, 140, 39 differentially abundant metabolites between the DSS and CON groups, between the B+DSS and CON groups, between the DSS and B+DSS groups were identified, respectively. Pathway analysis of these metabolites revealed that several pathways were affected in DSS-induced colitis, ranging from purine metabolism (aminoacyl-tRNA biosynthesis) to amino acid metabolism (such as alanine, aspartate, and glutamate metabolism, and arginine metabolism) (Supplementary Figure 3). These pathways were reported to contribute to intestinal inflammation in mice (Xie et al., 2021; Zhu et al., 2021). Among the 39 differentially abundant metabolites between the DSS and B+DSS groups, the trend of relative concentrations of 30 in the three groups showed “V” or “reverse-V” shape change (Figure 5D), indicating the effect of BAA2573 treatment. Therefore, we paid our attention mainly in the 39 metabolites (Figure 5C). Half of the 39 metabolites were lipids or organic oxygen compounds (Supplementary Table 1). KEGG pathway enrichment analysis was performed using these differentially abundant metabolites (Figure 5E). Starch and sucrose metabolism, vitamin B6 metabolism, sphingolipid metabolism, galactose metabolism, amino acid (glycine, serine, threonine, arginine, proline, and tyrosine) metabolism, and purine metabolism were the primary enriched pathways. Eight differentially abundant metabolites involved in the top eight pathways were further illustrated (Supplementary Figures 2C, D). Compared with the DSS group, the levels of trehalose, isomaltose, melezitose, and 4-Pyridoxic acid were significantly downregulated (*P* < 0.05). In contrast, the levels of sphingosine, creatine, L-trans-4-Methyl-2-pyrrolidinecarboxylic acid (L4M2P), and uric acid were significantly upregulated.

### 3.4. *Bifidobacterium longum* BAA2573 affected the microbiota–metabolite interactions in DSS-induced colitis mice

**Figure 6:**
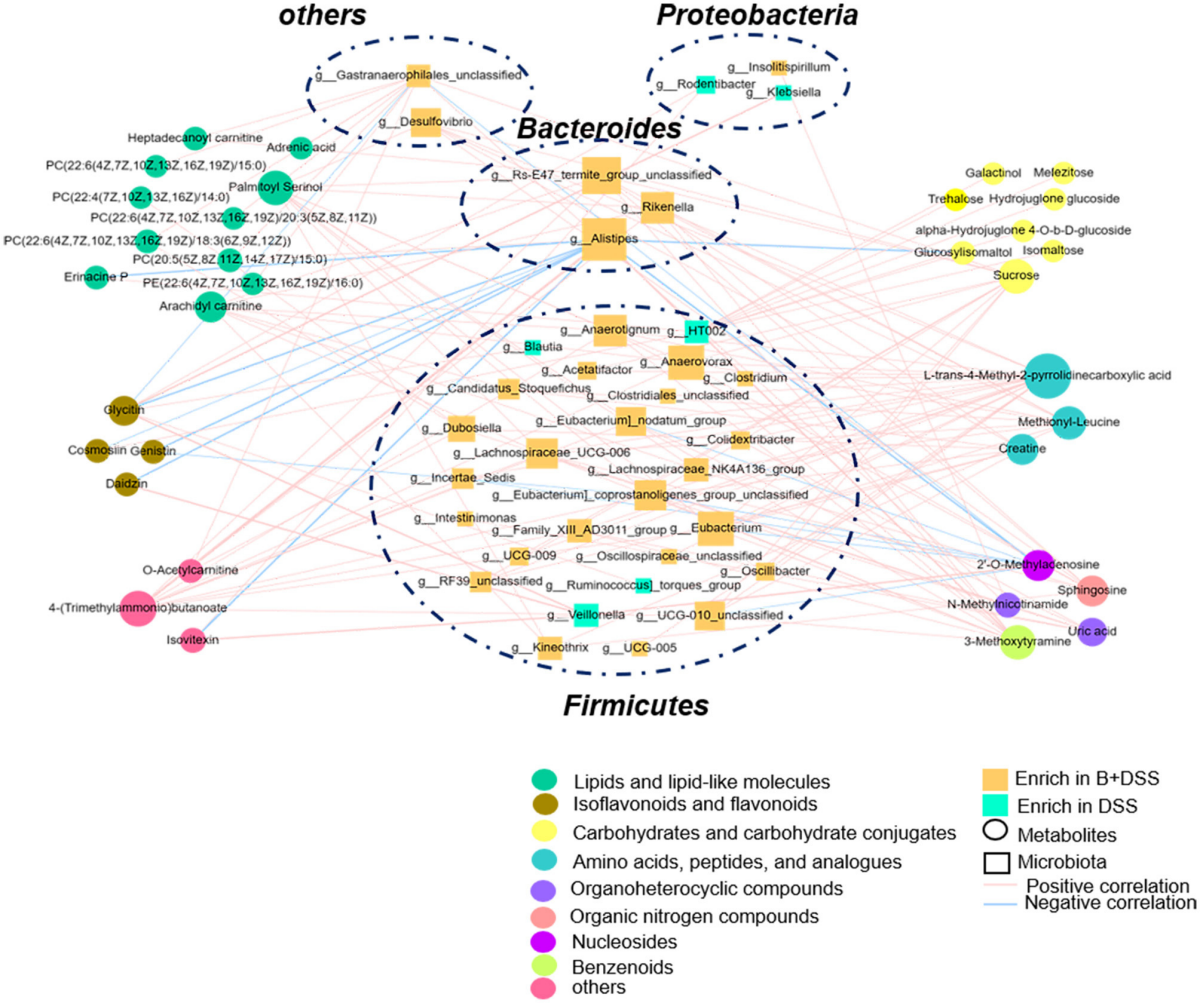
Interactions between differentially abundant microbiota and metabolite in the B+DSS and DSS groups (Pearson's correlation analysis, *r* > 0.4, *P* < 0.05).

To further identify the microbiota–metabolite interactions related to BAA2573 pretreatment, Pearson's correlation analysis was performed using 57 genera and 39 metabolites, and the main interplays are shown in a heatmap (Supplementary Figure 4). *g_Alistipes* was negatively correlated with eight metabolites (genistin, glycitin, daidzin, isovitexin, 2′-o-methyladenosine, erinacine P, cosmosiin, and glucosylisomaltol) and positively correlated with three metabolites [PC(20:5(5Z,8Z,11Z,14Z,17Z)/15:0), 4-(trimethylammonio)butanoate, and 3-methoxytyramine]. In addition, *g_Veillonella* and *g_Lachnospiraceae_NK4A136_group* both correlated positively with four metabolites. *g_Dubosiella* was positively correlated with five metabolites. These correlations are described in detail in Figure 6 (*r* > 0.4, *P* < 0.05). Correlation analysis revealed that *g_Alistipes* was likely to be the core genus, given its association with several substances. Notably, 2′-o-methyladenosine, cosmosiin, daidzin, erinacine P, and glycitin were related to different flora in opposite ways, highlighting the complexity of the gut microbiome. Other genera, including *g_Eubacrerium*, were also associated with metabolites and may be identified as potential biomarkers.

## 4. Discussion

In the present study, we collected fecal specimens to perform 16S rDNA sequencing and non-targeted metabolomic profiling after modeling and intervention, which could directly reflect the interplay between BAA2573 and gut flora. On comparison with the DSS group, we found that pretreatment with BAA2573 could alter microbial and metabolomic characterization in DSS-induced colitis (Figure 4) and reverse the dysbiosis of the gut commensal microorganisms, which manifested by the downregulation of harmful and opportunistic pathogens, such as *Klebsiella* and *Veillonella*, and the upregulation of beneficial genera, such as *Alistipes* and *Dubosiella*. On the contrary, the multiomics analysis marked the interaction between microbiota and metabolites. Possible mechanisms through which this happens might be through enhancing the enrichment of glycerophospholipids, fatty acyls, and amino acids in the B+DSS group and reducing the enrichment of carbohydrates, phenylpropanoids, and polyketides. It was assumed that BAA2573 might ameliorate colitis by improving microbial imbalance and further regulating metabolic pathways, thereby improving clinical symptoms. Previous studies also proved that pretreatment with diet and *Bifidobacterium longum* could improve acute DSS-induced colitis rather than ongoing chronic colitis, which highlighted the preventive therapeutic efficacy of prebiotics in IBD (Silveira et al., 2017). Clinically, IBD is a lifelong disease that often begins in childhood or adolescence. Early diagnosis and prompt management will improve the prognosis for PIBD (Oliveira and Monteiro, 2017), which is the reason we pretreated BAA2573 1 week before modeling to emphasize its adjuvant and preventive effects.

Previous studies had indicated that gut microbiota were deeply involved in the pathogenesis of IBD. Franzosa *et al*. found that the intestinal flora structure between the non-IBD group and the CD group was vastly different and that the heterogeneity in the UC group was higher (Franzosa et al., 2019). Nagalingam *et al*. and Munyaka et al. collected fecal specimens from murine colitis models and found that the microbiota abundance decreased after DSS treatment. The relative abundance of *Bacteroides* was decreased, while the relative abundance of Proteobacteria and Firmicutes was increased in DSS-induced colitis models, which was consistent with our experiment (Nagalingam et al., 2011; Munyaka et al., 2016). Inhibiting the overgrowth of harmful bacteria can significantly reduce colitis symptoms (Chen et al., 2022; Ma et al., 2022). As a member of the Enterobacteriaceae family in Proteobacteria phylum, *Klebsiella* was mostly harmful and abundant in the DSS group. In the B+DSS group, the relative abundance of *Klebsiella* decreased significantly after pretreatment with BAA2573 (Figure 6), which was consistent with a previous study with another *Bifidobacterium* subspecies (Fan et al., 2021). *Klebsiella* with pathogen-associated molecular patterns (PAMPs) could be identified by the toll-like receptor (TLR) family (like TLR2/4) and promote intestinal inflammation (Chalifour et al., 2004).

Especially, flora may be used as markers to predict response to treatment or prognosis. *Veillonella*, the opportunistic pathogens, is one of the few species whose abundance sharply decreased in *the* Firmicutes phylum after BAA2573 intervention, indicating that *Veillonella* may play a harmful role in colitis, and the underlying mechanisms need to be further explored. The abundance of *Veillonella* was significantly elevated in patients with CD (Pittayanon et al., 2020) and exhibited a strong immune response to serum IgG (Bourgonje et al., 2022), which may be associated with the immune tolerance of colonized bacteria in the gastrointestinal tract. Shaw *et al*. found that *Veillonella* was one of the differentiated microbiota at the genus level between therapeutic responders and non-responders in PIBD (Shaw et al., 2016). In adult IBD patients complicated with primary sclerosing cholangitis, the abundance of *Veillonella* was significantly increased. Moreover, *Veillonella* also played a differentiating role in IBD-related liver disease (Kummen et al., 2017).

In addition to the abovementioned downregulated species, *Alistipes* and *Dubosiella, Candidatus_Saccharimonas, Lachnospiraceae_NK4A136_group*, and *Oscillibacter* had significantly increased after BAA2573 intervention, which may participate in the recovery of colitis, and the results are consistent with previous studies (Hao et al., 2021; Wan et al., 2022). *Alistipes*, a relatively new member of *Bacteroides*, was isolated primarily from clinical samples and participated in chronic disease (Parker et al., 2020). Meat-based diet could increase the abundance *Alistipes* (David et al., 2014), but it was negatively correlated with serum triglyceride levels (Liu Liu X. et al., 2022). Lipid metabolism disorders are involved in intestinal inflammation, suggesting *Alistipes* was closely related to lipid metabolism and gut health (Wu et al., 2022). In our study, *Alistipes* was significantly correlated with lipids and lipid-like molecules in feces, including Erinacine P and PC(20:5(5Z,8Z,11Z,14Z,17Z)/15:0). Therefore, BAA2573 may increase the abundance of *Alistipes* and improved colitis by modulating the lipid metabolic process. Interestingly, *Dubosiella, Lachnospiraceae_NK4A136_group, Oscillibacter*, and *Alistipes* belong to the phylum Firmicutes and are producers of short-chain fatty acids (SCFAs), especially butyric acid (Parada Venegas et al., 2019; Yuan et al., 2022). As the main energy source of colonocytes, butyric acid has been proven to play an indispensable role in relieving IBD symptoms by reducing inflammation (Li et al., 2021), strengthening epithelial barrier (Chen et al., 2018), and modulating immunity (Liu et al., 2020). However, we did not observe a significant increase in SCFAs in fecal specimens, probably because of the methods or samples we used. Detection methods such as SCFA targeted profiling with fecal specimens or serum may be needed to determine changes in SCFAs.

In addition to the gut microbiota, metabolites also participate in maintaining intestinal homeostasis. KEGG enrichment analysis with differentially abundant metabolites showed that pretreatment with BAA2573 could improve carbohydrate metabolism, mainly the glucose metabolism pathway. The levels of trehalose, isomaltose, and melezitose were significantly downregulated. In correspondence to our finding, recent studies showed that probiotics containing *Bifidobacterium longum* could reduce intestinal inflammation caused by Enterotoxigenic *Escherichia coli* (ETEC) by balancing enteric microorganism and improving carbohydrate metabolism (Li et al., 2022). At the brush edge of small intestinal epithelial cells, trehalose is broken down into glucose, which is transported into cells and provides energy via glycolytic reaction (d'Enfert et al., 1999), so trehalose is consumed by the small intestine and expressed at low levels in the colon. In the present study, *Alistipes*, the butyrate-producing bacteria was significantly negatively correlated with the abundance of trehalose (Portincasa et al., 2022). Therefore, we speculated that BAA2573 might promote the growth of beneficial bacteria and further consume intestinal carbohydrates to provide energy. On the contrary, metabolites produced by these beneficial bacteria, such as butyrate, may contribute to ameliorating colitis. Another investigation showed that high-sugar diet induced changes in the microbiota of the mouse, leading to a decrease the abundance of *Bacteroides* (Do et al., 2018). Therefore, *Alistipes* and trehalose could be interdependent, with a mutual effect in intestinal microecology.

The Vitamin B6 metabolic pathway takes effect in IBD. Low serum levels of vitamin B6 were common in IBD patients (MacMaster et al., 2021). Vitamin B6 deficiency could result in hyperhomocysteinemia (Hhcy), an aggravated colon inflammation in mice. Dietary supplementation of vitamin B6 could reduce the inflammatory indexes of colitis in mouse models (Selhub et al., 2013; Flannigan et al., 2014). 4-Pyridoxic acid (4-PA) is one of the main metabolites of vitamin B6 (Stover and Field, 2015). In humans, a high level of 4-PA in serum was positively correlated with high colorectal cancer risk and high mortality in type 2 diabetes mellitus (T2DM) (Xu et al., 2022; Zhang et al., 2022). Conspicuously, the high level of 4-PA has caused certain damage to the intestine, but it was noteworthy that, after pretreatment with BAA2573, the level of 4-PA was remarkably downregulated. Therefore, the level of 4-PA may be an indicator of the severity of IBD. Vitamin B6 supplement could be another research target when we further explore the underlying mechanism.

According to the correlation analysis of microbes and metabolites in our study, *Alistipes* tends to be the central positional indicator. It was negatively correlated with isoflavones, including glycitin and daidzin. In contrast, *Veillonella* was positively correlated with isoflavones, which were both downregulated in the B+DSS group. Previous studies reported that a diet with isoflavones showed an anti-inflammatory effect in mice (Shrode et al., 2022). Glycitin tended to inhibit inflammation via the nuclear factor-kappa B (NFκB) or mitogen-activated protein kinase (MAPK) pathway (Chen Y. et al., 2019; Wang et al., 2020). On the contrary, isoflavones are structurally similar to 17-β-estradiol and bind to estrogen receptors (ERα and ERβ), participating in regulating the effects of estrogen in humans (Kim, 2021). *Bifidobacterium longum*-a and *Veillonella* sp. strain EP were reported to convert daidzin to daidzein and then transformed daidzein to equol in the colon (Rafii, 2015). In the urine of sporadic colorectal adenomas patients, the levels of equol were significantly lower (Polimeno et al., 2020). We speculated that isoflavones, such as daidzin and glycitin, may be degraded by anaerobic bacteria in the colon (Park et al., 2011) and play anti-inflammatory and hyperplasia-inhibiting roles. In summary, we could further detect the production of equol for the elaboration of specific mechanisms of isoflavones on IBD and its relationship to gut flora.

To our knowledge, sphingosine could be transformed to sphingosine-1-phosphate (S1P) by sphingosine kinase (Tsai and Han, 2016). In active UC patients, the level of S1P in plasma was upregulated, and higher enrichment of *Klebsiella* in fecal revealed a positive connection with S1P (Sun et al., 2019). Animal experiments had detected that disturbing the sphingolipid metabolism could improve the colorectal tumor microenvironment and reduce the severity of UC in mice (Lv et al., 2019; Jiang et al., 2021). Sphingosine kinase and S1P receptors may become an emerging therapeutic target or predictive markers of therapeutic response in IBD (Sukocheva et al., 2020; Elhag et al., 2022). In the present study, the level of sphingosine, which was positively correlated with the relative abundance of *Lachnospiraceae_NK4A136_group* and *Lachnospiraceae_UCG-006*, was downregulated after model establishment and reversed after the intervention of BAA2573, indicating that BAA2573 possibly inhibited the activity of sphingosine kinase and reduced the level of S1P in the gut during the disease healing process. Further research will be conducted to fully understand the mechanisms involved.

## 5. Limitations of this study

Our study has a few limitations. First, in this animal experiment, we described the protective effect of pretreatment of *Bifidobacterium longum* BAA2573 on DSS-induced colitis and correlations between abundant microbiota and metabolites, which did not provide sufficient causality verification. Therefore, further *in vivo* or *in vitro* functional studies are needed to identify the relationship between microbiota, metabolites, and the healing of colitis. Second, as representative components of SCFAs and the research emphasis in gut microbiota, substances like acetic acid and butyric acid were not identified significant difference in our study. Therefore, expanding the study by performing targeted metabolomics analysis of urinal or serum samples may be benefited by further in-depth study. Third, to better embody the advantages of *Bifidobacterium longum* in the treatment of IBD, we should pit single BAA2573 against BAA2573 combined with recent medications on DSS-induced colitis mice, such as 5-aminosalicylic acid or other single species of probiotics.

## 6. Conclusion

Our study presented functional insights of a single substance from a widely used probiotic mixture by establishing an animal model of colitis and introducing 16S rDNA sequencing and non-targeted metabolomic profiling, which demonstrated that the symptom of colitis was improved, the inflammation of the colon was alleviated, and the gut microbiome and metabolites were altered in the B+DSS group compared to the DSS group. The application of *Bifidobacterium longum* BAA2573 as a new probiotic deserves further research and clinical verification.

## Data availability statement

The 16s sequence data in the present study were deposited in the NCBI repository with accession number PRJNA962613. The untargeted metabolomic profiling is available in Metabolights (https://www.ebi.ac.uk/metabolights/MTBLS7751).

## Ethics statement

The animal study was reviewed and approved by the Ethics Committee of Wuxi Children's Hospital.

## Author contributions

QL and W-JH designed the study, participated in most experimental work, analyzed the results, and edited the manuscript. X-YW, R-MZ, and C-LH performed the experiments and acquired results. Y-SL and X-ZL directed the experiment and revised the manuscript. All authors read and approved the manuscript.
