# Supplementary material for: Pretreatment with Bifidobacterium longum BAA2573 ameliorates dextran sulfate sodium (DSS)-induced colitis by modulating gut microbiota
Source: Front Microbiol. 2023 Jun 6;14:1211259. doi: 10.3389/fmicb.2023.1211259 (PMC10280014; doi:10.3389/fmicb.2023.1211259)
Supplement: Supplementary file 1 [file Data_Sheet_1.pdf]

Supplementary Material

Pretreatment with *Bifidobacterium longum* BAA2573 ameliorates dextran sulfate sodium (DSS)-induced colitis by modulating gut microbiota.

Qiong Lin[1,2]†, Wu-Juan Hao[2]†, Ren-Min Zhou[2], Cui-Lan Huang[3], Xu-Yang Wang[3], Yan-Shan Liu[4]*, Xiao-Zhong Li[1]*

*** Correspondence:** Yan-Shan Liu, E-mail:liu_yshan@hotmail.com; Xiao-Zhong Li,E-mail: xiaozhonglicn@yeah.net.

1.1 Supplementary Figures

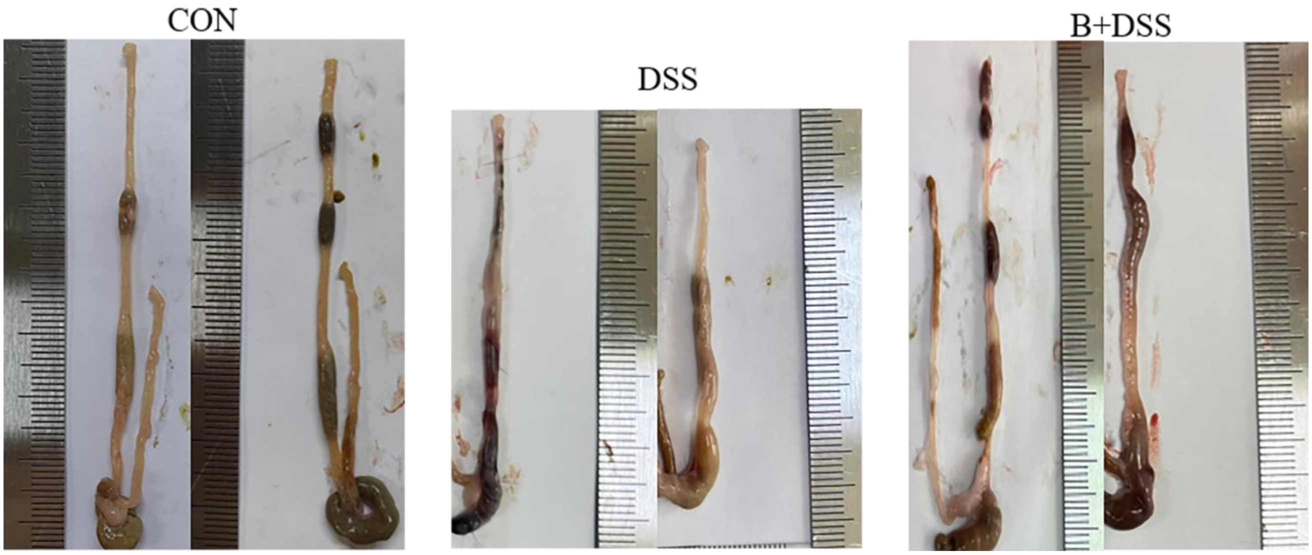

Supplementary Figure 1. Photos of colon tissue

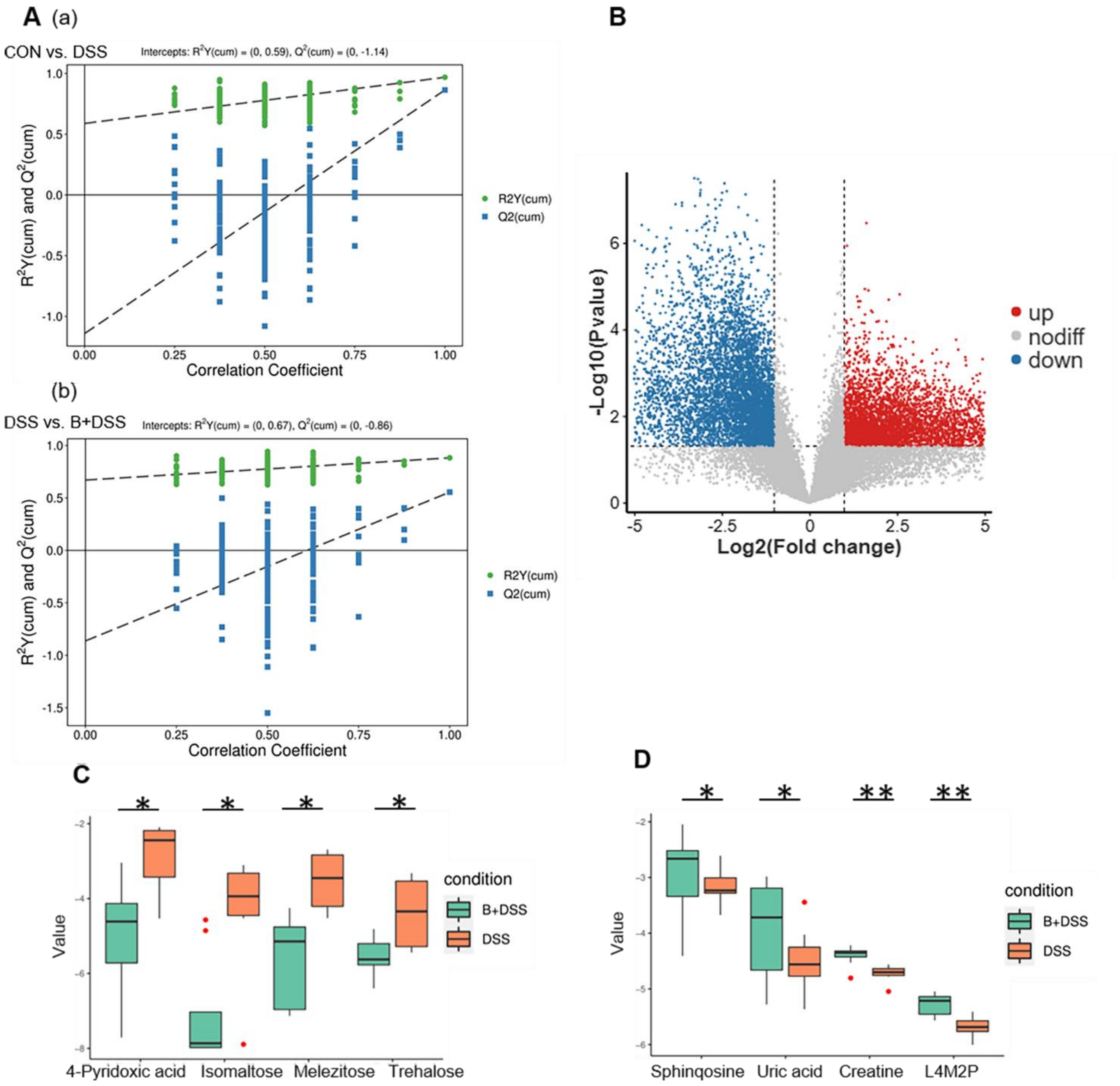

Supplementary Figure 2 Pretreatment of BAA2753 altered metabolites of colon in DSS-induced colitis. **(A)** Permutation test show the good validity of OPLS-DA model. The intercepts of R^2= (0.0, 0.59) and Q^2= (0.0,–1.14), R^2= (0.0, 0.67) and Q^2= (0.0,–0.86), suggest that the OPLS-DA model is not overfitting. **(B)** Volcano plots showing the results of comparisons of metabolites in CON group relative to DSS group. The vertical dashed lines indicate the threshold for the twofold abundance difference. The horizontal dashed line indicates the p=0.05 threshold. Comparisons between two groups were performed using Student's t-test. Metabolites with significant changes are presented in red (upregulated) or Blue (downregulated). (**C,D**) Results of statistical analysis in differentially abundant metabolites. Y-axis value equal to Log10 (relative abundance of each metabolite). The data are expressed as the means ± SDs (n = 8). *P < 0.05. **P < 0.01.

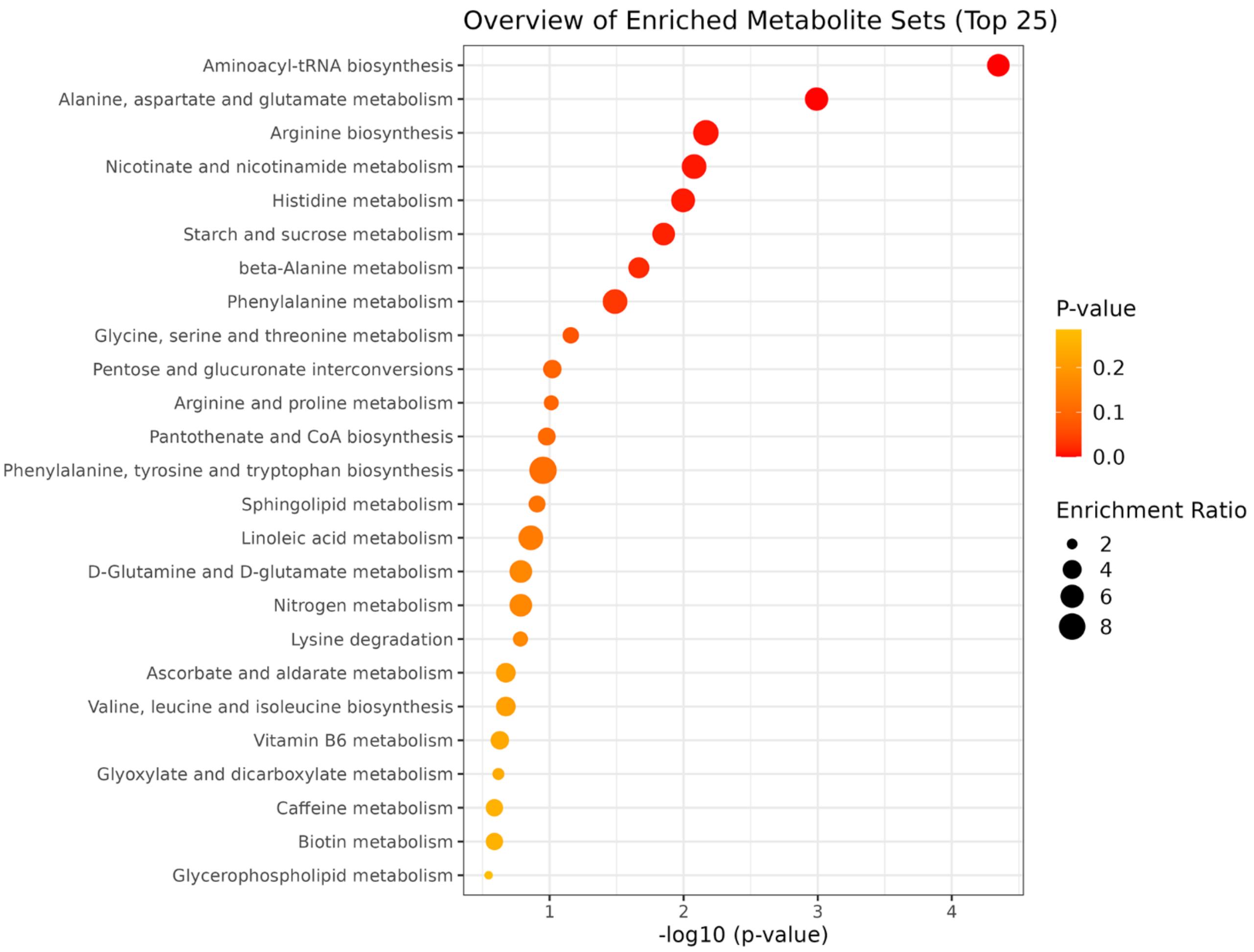

Supplementary Figure 3 Pathway enrichment analysis of significantly abundant metabolites in DSS group and CON group according to the KEGG pathway.

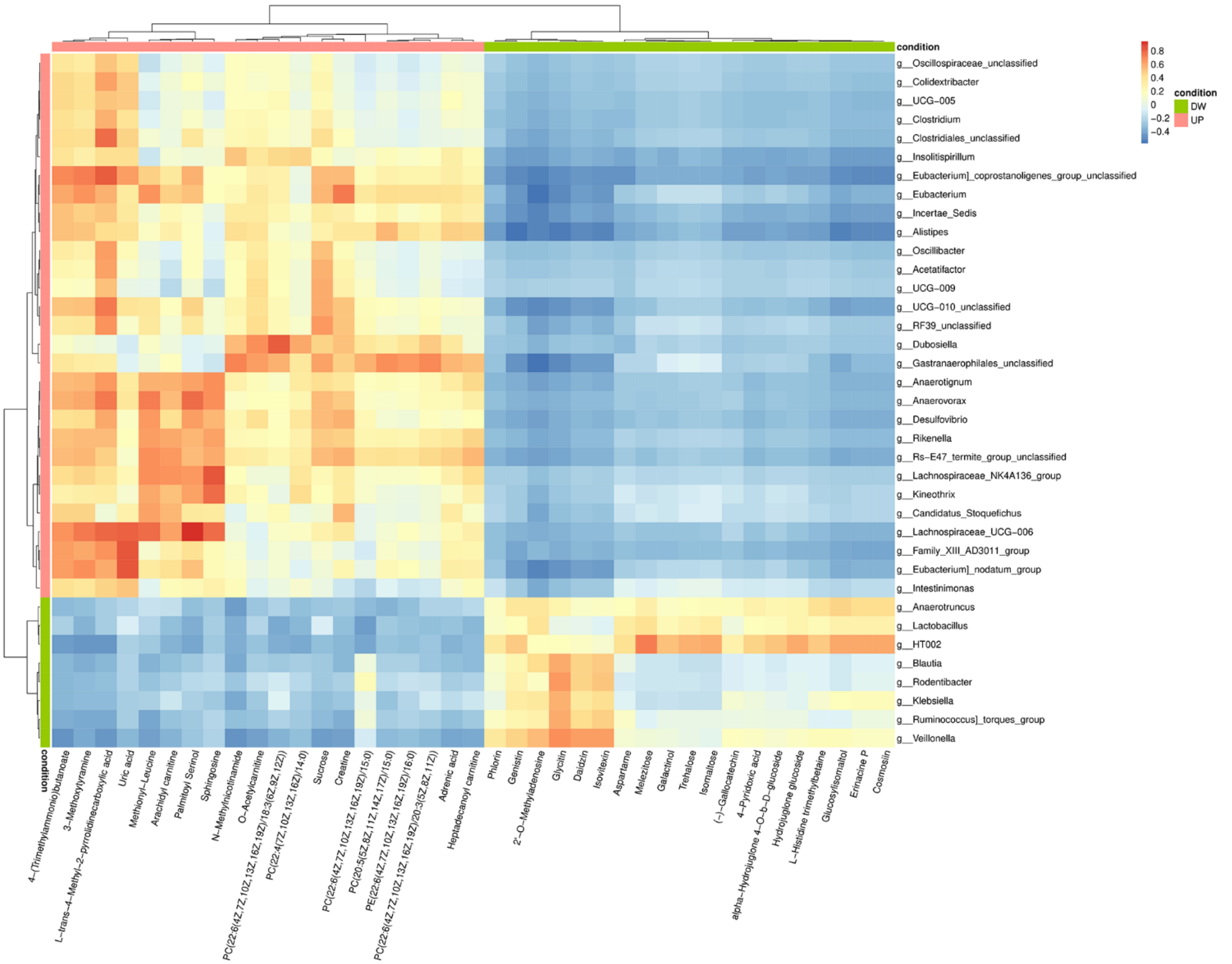

Supplementary Figure 4. Heatmap illuminates main interplays between differentially abundant microbiota and metabolites. Their conditions, such as up-regulated or down-regulated, in B+DSS group are also shown in diagram.

1.2 Supplementary Tables

Supplementary Table 1 Differentially abundant metabolites between DSS and B+DSS groups

Class	Name	P-value	Log2(Foldchange)
Benzenoids	3-Methoxytyramine	0.0047	1.9277
Lipids and lipid-like molecules	Erinacine P	0.0069	-5.6270

Lipids and lipid-like molecules	PC(22:6(4Z,7Z,10Z,13Z,16Z,19Z)/15:0)	0.0225	1.1458
Lipids and lipid-like molecules	Arachidyl carnitine	0.0410	1.5762
Lipids and lipid-like molecules	PC(22:6(4Z,7Z,10Z,13Z,16Z,19Z)/18:3(6Z,9Z,12Z))	0.0149	1.6473
Lipids and lipid-like molecules	Palmitoyl Serinol	0.0271	1.8043
Lipids and lipid-like molecules	PC(20:5(5Z,8Z,11Z,14Z,17Z)/15:0)	0.0243	1.8136
Lipids and lipid-like molecules	Heptadecanoyl carnitine	0.0446	1.8720
Lipids and lipid-like molecules	PC(22:6(4Z,7Z,10Z,13Z,16Z,19Z)/20:3(5Z,8Z,11Z))	0.0251	2.0610
Lipids and lipid-like molecules	Adrenic acid	0.0193	2.1401
Lipids and lipid-like molecules	PC(22:4(7Z,10Z,13Z,16Z)/14:0)	0.0297	2.5695
Lipids and lipid-like molecules	PE(22:6(4Z,7Z,10Z,13Z,16Z,19Z)/16:0)	0.0336	2.6025
Nucleosides, nucleotides, and analogues	2'-O-Methyladenosine	0.0053	-4.3252
Organic acids and derivatives	L-Histidine trimethylbetaine	0.0221	-4.3980
Organic acids and derivatives	Aspartame	0.0101	-3.4681
Organic acids and derivatives	Creatine	0.0021	1.0845

Organic acids and derivatives	Methionyl-Leucine	0.0276	1.2070
Organic acids and derivatives	L-trans-4-Methyl-2-pyrrolidinecarboxylic acid	0.0042	1.3497
Organic nitrogen compounds	Sphingosine	0.0183	1.937581829
Organic oxygen compounds	Melezitose	0.0307	-5.8120
Organic oxygen compounds	Isomaltose	0.0367	-5.7369
Organic oxygen compounds	Trehalose	0.0431	-5.1289
Organic oxygen compounds	Glucosylisomaltol	0.0071	-4.5277
Organic oxygen compounds	Phlorin	0.0178	-4.3564
Organic oxygen compounds	Hydrojuglone glucoside	0.0226	-3.6491
Organic oxygen compounds	Galactinol	0.0351	-3.5648
Organic oxygen compounds	alpha-Hydrojuglone 4-O-b-D-glucoside	0.0231	-3.5018
Organic oxygen compounds	Sucrose	0.0096	2.0920
Organoheterocyclic compounds	4-Pyridoxic acid	0.0185	-4.4413
Organoheterocyclic compounds	N-Methylnicotinamide	0.0071	1.2601

Organoheterocyclic compounds	Uric acid	0.0413	1.5257
others	Isovitexin	0.0226	-4.9128
others	(-)-Gallocatechin	0.0340	-3.5440
others	O-Acetylcarnitine	0.0070	1.6350
others	4-(Trimethylammonio)butanoate	0.0021	1.8643
Phenylpropanoids and polyketides	Genistin	0.0065	-5.7849
Phenylpropanoids and polyketides	Cosmosiin	0.0153	-5.3861
Phenylpropanoids and polyketides	Daidzin	0.0243	-5.3317
Phenylpropanoids and polyketides	Glycitin	0.0161	-4.6122

1.3 Data Availability Statement

The 16s sequence data in the present study were deposited in the NCBI repository with accession number PRJNA962613. The untargeted metabolomic profiling is available in Metabolight (https://www.ebi.ac.uk/metabolights/MTBLS7751).
